# Cardiorespiratory synchronisation and systolic blood pressure correlation of peripheral arterial stiffness during endoscopic thoracic sympathectomy

**DOI:** 10.1038/s41598-021-85299-y

**Published:** 2021-03-16

**Authors:** Toshifumi Muneyasu, Harutoyo Hirano, Akira Furui, Zu Soh, Ryuji Nakamura, Noboru Saeki, Yoshiyuki Okada, Masashi Kawamoto, Masao Yoshizumi, Atsuo Yoshino, Takafumi Sasaoka, Shigeto Yamawaki, Toshio Tsuji

**Affiliations:** 1 Graduate School of Advanced Science and Engineering, Hiroshima University, 1-4-1 Kagamiyama, Higashi-Hiroshima, Hiroshima 739-8527 Japan; 2 College of Engineering, Academic Institute, Shizuoka University, 3-5-1, Johoku, Naka-ku, Hamamatsu, Shizuoka 432-8561 Japan; 3 Department of Anesthesiology and Critical Care, Graduate School of Biomedical and Health Sciences, Hiroshima University, 1-2-3 Kasumi, Minami-ku, Hiroshima, Hiroshima 734-8553 Japan; 4 Department of Special Care Dentistry, Graduate School of Biomedical and Health Sciences, Hiroshima University, 1-2-3 Kasumi, Minami-ku, Hiroshima, Hiroshima 734-8553 Japan; 5 Medical Corporation JR Hiroshima Hospital, 3-1-36 Futabano-sato, Higashi-ku, Hiroshima, Hiroshima 732-0057 Japan; 6 Department of Cardiovascular Physiology and Medicine, Graduate School of Biomedical and Health Sciences, Hiroshima University, 1-2-3 Kasumi, Minami-ku, Hiroshima, Hiroshima 734-8553 Japan; 7 Department of Psychiatry and Neurosciences, Graduate School of Biomedical and Health Sciences, Hiroshima University, 1-2-3 Kasumi, Minami-ku, Hiroshima, Hiroshima 734-8551 Japan; 8 Center for Brain, Mind and KANSEI Sciences Research, Hiroshima University, 1-2-3 Kasumi, Minami-ku, Hiroshima, Hiroshima 734-8551 Japan

**Keywords:** Biomarkers, Predictive markers, Cardiovascular biology, Neurophysiology, Respiration

## Abstract

Muscle sympathetic nerve activity (MSNA) is known as an effective measure to evaluate peripheral sympathetic activity; however, it requires invasive measurement with the microneurography method. In contrast, peripheral arterial stiffness affected by MSNA is a measure that allows non-invasive evaluation of mechanical changes of arterial elasticity. This paper aims to clarify the features of peripheral arterial stiffness to determine whether it inherits MSNA features towards non-invasive evaluation of its activity. To this end, we propose a method to estimate peripheral arterial stiffness $\beta$ at a high sampling rate. Power spectral analysis of the estimated $\beta$ was then performed on data acquired from 15 patients ($23.7 \pm 9.0$ years) who underwent endoscopic thoracic sympathectomy. We examined whether $\beta$ exhibited the features of MSNA where its frequency components synchronise with heart and respiration rates and correlates with the low-frequency component of systolic blood pressure. Regression analysis revealed that the local peak frequency in the range of heartbeat frequency highly correlate with the heart rate ($R^{2}=0.85$, $p=6.3\times 10^{-13}$) where the regression slope was approximately 1 and intercept was approximately 0. Frequency analysis then found spectral peaks of $\beta$ approximately 0.2 Hz that correspond to the respiratory cycle. Finally, cross power spectral analysis showed a significant magnitude squared coherence between $\beta$ and systolic blood pressure in the frequency band from 0.04 to 0.2 Hz. These results indicate that $\beta$ inherits the features observed in MSNA that require invasive measurements, and thus $\beta$ can be an effective non-invasive substitution for MSNA measure.

## Introduction

The autonomic nervous system consists of the sympathetic and parasympathetic nervous system, and the balance between these systems regulates the physical and mental state.

Numerous methods for evaluating autonomic nervous activity from various physiological responses have been proposed using information derived from heart rate^1^, respiration^2^, electroencephalography^3^, pupil^4^, and skin temperature^5^. Heart rate variability (HRV) analysis is an easy and representative method to evaluate autonomic nervous activity^1^. This method focuses on the periodic fluctuations present in heartbeats. The low frequency (LF) and high frequency (HF) components in HRV are known to synchronise with cardiac sympathetic/parasympathetic nervous activity and cardiac parasympathetic nervous activity, respectively^1^. This method, however, evaluates only cardiac autonomic nervous activity and cannot evaluate peripheral autonomic nervous activity.

Microneurography has been proposed as a representative method for evaluating peripheral autonomic nervous activity^6^. In this method, muscle sympathetic nerve activity (MSNA) can be measured directly by inserting a needle electrode into the unanesthetised leg or arm percutaneously. Microneurography, however, has a limitation for measuring MSNA as this method requires an invasive procedure, which places additional strain on subjects. Meanwhile, our research group proposed the peripheral arterial stiffness as an index to noninvasively evaluate peripheral autonomic nervous activity^7,8^. We also showed that quantitative evaluation of human pain sensation was possible with the peripheral arterial stiffness^8^.

The MSNA has a close relationship with peripheral arterial stiffness in regulating blood pressure by contracting vascular smooth muscles^9^, and the peripheral arterial stiffness reflects this contraction. Therefore, peripheral arterial stiffness may exhibit features of MSNA. Previous studies reported that the frequency components of MSNA contain information about vital signals: the approximately 1 Hz^10^ of MSNA synchronise with heartbeat, the 0.15–0.4 Hz component synchronise with respiration rhythm^11^, and the 0.04–0.15 Hz component correlates with systolic blood pressure^11^. The MSNA thus may have a potential to be noninvasively evaluated using the peripheral arterial stiffness if it preserves the above-mentioned components. However, this method can only estimate the peripheral arterial stiffness based on heartbeat^7,8^, and this sampling frequency is insufficient to determine whether peripheral arterial stiffness preserves the periodic components in MNSA.

Against the above background, this paper aims to extract the frequency information embedded in peripheral arterial stiffness. To this end, a novel method to estimate peripheral arterial stiffness^7^ at a high frequency was developed. The proposed method is applied to data measured during endoscopic thoracic sympathectomy surgery because it includes clear-cut fluctuations in the state of sympathetic nerve activity. Power spectral analysis of peripheral arterial stiffness was then carried out to reveal the relationship between each frequency component and the vital signals.

**Figure 1:**
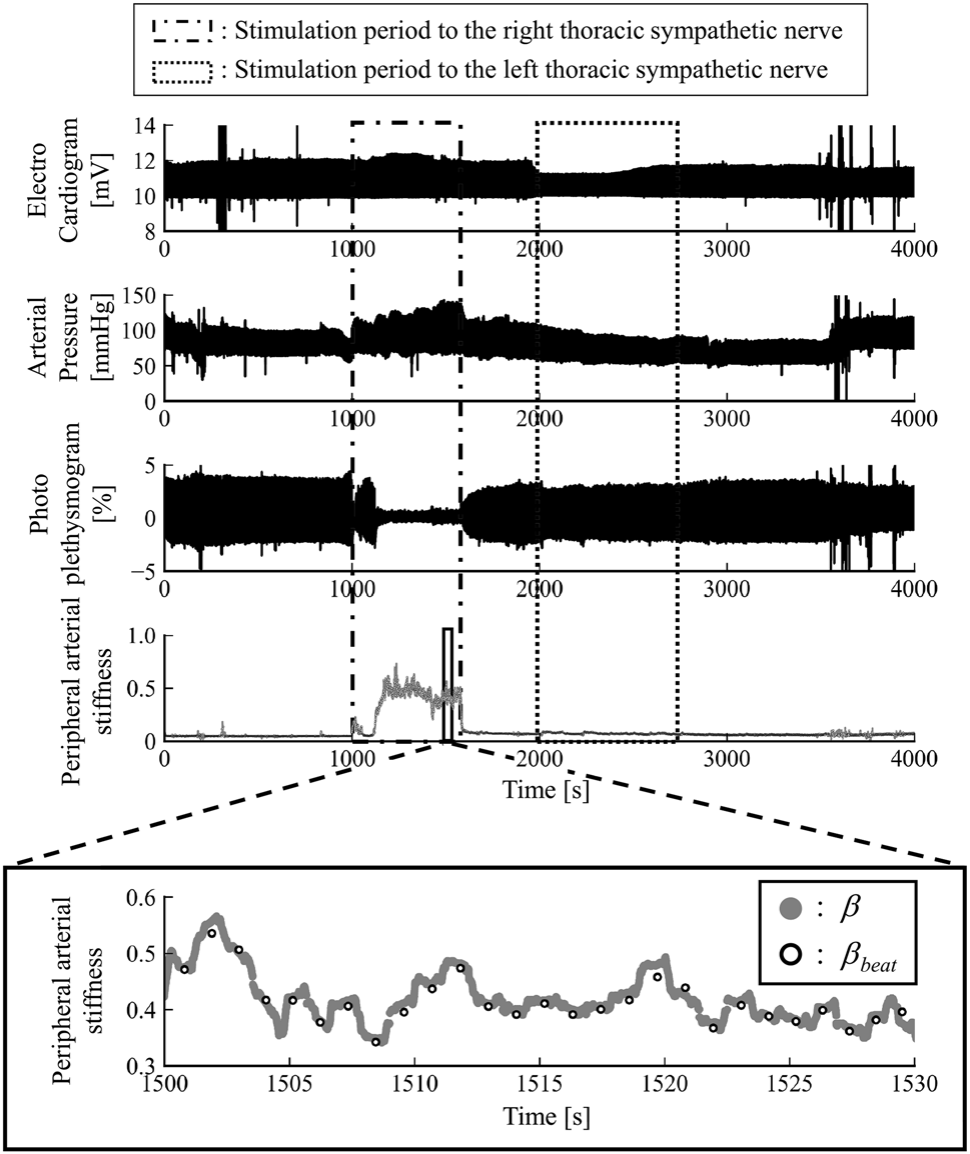
Measured biological signals. From top to bottom, electrocardiograms, continuous arterial pressures, photo plethysmograms, proposed peripheral arterial stiffness $\beta$, and conventional peripheral arterial stiffness $\beta _{beat}$ from Sub. A are shown.

## Methods

### Experimental configurations

Endoscopic thoracic sympathectomy (ETS) is a surgical procedure for the treatment of hyperhidrosis^12^. The ETS improves sweating by clipping off the sympathetic trunk near the thoracic spine. Peripheral sympathetic nerve activity decreases after clipping the sympathetic nerve, which may reduce the response of both MSNA and peripheral arterial stiffness. Therefore, the author selected adult patients undergoing ETS as subjects. In this paper, biological signals of subjects who underwent the T3 level of ETS in the order of right and left parts were analysed. During surgery, the subjects underwent general anaesthesia and the respiratory rate was set at approximately 12 times/min (0.2 Hz) using a ventilator. Electrocardiogram, invasive radial arterial blood pressure from a catheter inserted into the right radial artery, and a photoplethysmogram from the thumb on the right hand were measured using a bedside monitor (BSS-9800, Nihon Kohden Corp., Tokyo, Japan). The measured data was stored digitally at a sampling frequency of 125 Hz.

Data from fifteen patients (five male and ten female patients, mean age ± S.D. $23.7 \pm 9.0$ years.) were analysed in this paper. Experiments were conducted in accordance with the Declaration of Helsinki. Informed consent was obtained from all study subjects before the experiments were performed, and the study was approved by the Hiroshima University Ethics Committee.

**Figure 2:**
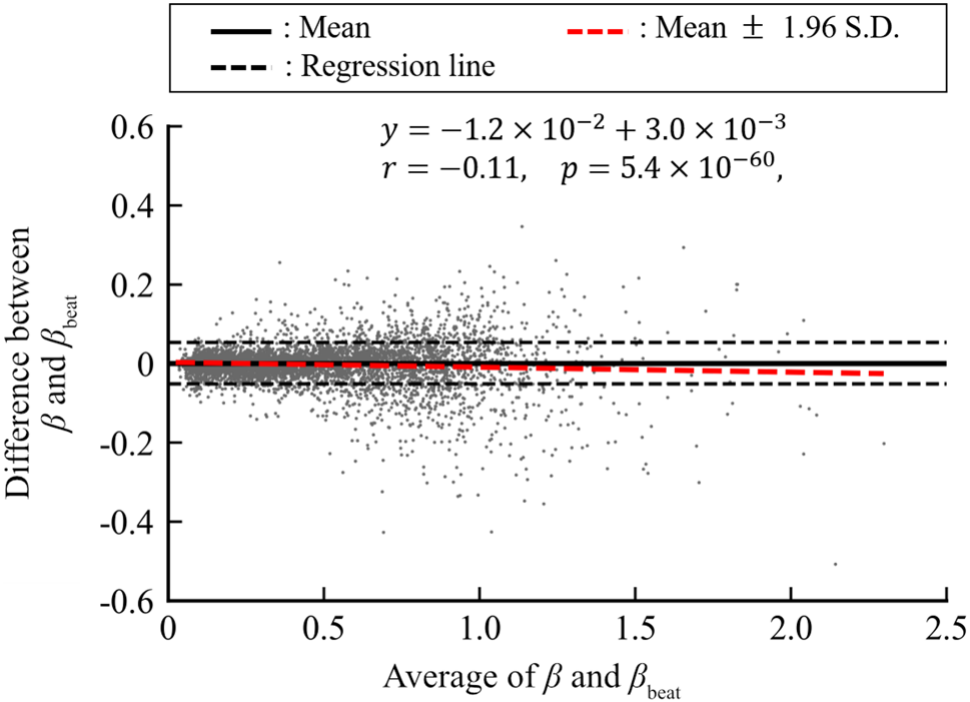
Bland–Altman plot between proposed peripheral arterial stiffness $\beta$ and conventional peripheral arterial stiffness $\beta _{beat}$.

### Proposed estimation method of peripheral arterial stiffness

Peripheral arterial stiffness was estimated from the measured electrocardiogram, continuous blood pressure, and photoplethysmogram using the following log-linearised peripheral arterial viscoelastic model^7,8^.

1 $$\begin{aligned} P_{b}(t)= \, & {} \mu {\ddot{P}}_{l}(t)+\eta {\dot{P}}_{l}(t) +\exp \{ \beta P_{l}(t)+P_{b\beta _{0}}+P_{b\beta _{nl}}(P_{l}(t))\}, \end{aligned}$$

where $\mu$, $\eta$ and $\beta$ are the arterial wall inertia, viscosity and stiffness, respectively. ${P_b}(t)$, ${P_l}(t)$, $\dot{P_l}(t)$ and $\ddot{P_l}(t)$ are the arterial blood pressure at the time *t*, photoplethysmogram, its change rate and its acceleration, respectively. $P_{b\beta _{0}}$ represents the reference blood pressure, and $P_{b\beta _{nl}}(P_{l}(t))$ represents the venous pressure component.

Conventionally, peripheral arterial stiffness is estimated for each heart-beat using the data measured in each R-R interval (refer to Supplemental Information S1). In contrast, this study estimates the peripheral arterial stiffness for each sampling time using the following procedure. First, the exponential term in Eq. (1) is approximated linearly by a Maclaurin series expansion around $P_{l}(t)=0$, as follows:

2 $$\begin{aligned} P_{b}(t)\approx \, & {} \mu {\ddot{P}}_{l}(t)+\eta {\dot{P}}_{l}(t) + C_{1}\exp \{ P_{b\beta _{0}}+P_{b\beta _{nl}}(0) \}P_{l}(t) + \exp \{P_{b \beta _{0}}+P_{b \beta _{nl}}(0) \}, \end{aligned}$$

where $C_{1}$ is defined as follows:

$$\begin{aligned} C_{1}= \, & {} \beta + \left. \frac{dP_{b\beta _{nl}}(P_{l}(t))}{dP_{l}(t)} \right| _{P_{l}(t) = 0}. \end{aligned}$$

Then, $\mu$, $\eta$, $C_{1}\exp \{ P_{b\beta _{0}}+P_{b\beta _{nl}}(0) \}$ and $\exp \{P_{b \beta _{0}}+P_{b \beta _{nl}}(0) \}$ are estimated by performing the linear least squares method on the blood pressure ${P_b}(t)$ and photoplethysmogram ${P_l}(t)$ for arbitrary window length *w* using Eq. (2).

Second, the estimated inertia $\mu$ and viscosity $\eta$ are substituted into Eq. (1). Taking the exponent on both sides of that yields the following equation:

3 $$\begin{aligned} \beta P_{l}(t) + P_{b\beta _{0}}+P_{b\beta _{nl}}(P_{l}(t))={\text{ln}} \{ P_{b}(t)-\mu {\ddot{P}}_{l}(t)-\eta {\dot{P}}_{l}(t) \}. \end{aligned}$$

Stiffness $\beta$ is estimated within the range that the blood pressure is higher than the mean blood pressure so that venous pressure component can be ignored. Then, $P_{b\beta _{nl}}(P_{l}(t))$ becomes zero. $\beta$ and $P_{b\beta _{0}}$ are estimated based on Eq. (3) using the linear least squares method with the blood pressure ${P_b}(t)$ and photoplethysmogram ${P_l}(t)$ for arbitrary window at a length of *w*.

By repeating the above procedure while shifting the data for window *w* each sampling time, the peripheral arterial stiffness $\beta$ can be estimated as equally spaced data as a sampling time. In this paper, the peripheral arterial stiffness $\beta$ was estimated by setting the window length *w* to the maximum heartbeat interval of each subject.

### Analysis

#### Accuracy verification of the estimation of peripheral arterial stiffness

The peripheral arterial stiffness $\beta$ estimated by the proposed method was compared with the peripheral arterial stiffness $\beta _{beat}$ estimated by the conventional method^7,8^ to verify the estimation accuracy. Whereas the proposed peripheral arterial stiffness $\beta$ was estimated at a sampling frequency of 125 Hz, the conventional peripheral arterial stiffness $\beta _{beat}$ was estimated for each heartbeat. The proposed peripheral arterial stiffness $\beta$ was resampled at each heartbeat after anti-aliasing processing using a low-pass filter with a cutoff frequency of 0.5 Hz. Correlation analysis was then performed between resampled proposed peripheral arterial stiffness $\beta$ and $\beta _{beat}$ obtained from the conventional method. Bland–Altman analysis^13^ was performed to compare absolute errors between the peripheral arterial stiffness $\beta$ and $\beta _{beat}$. The estimated $\beta$, along with other parameters, was then substituted into Eq. (2) so that the blood pressure predicted by the model could be obtained. The predicted blood pressure was then compared to the measured blood pressure for verification. The stiffness $\beta$ and $\beta_{\mathrm{{beat}}}$ were adopted only for data, where the coef?cient of determination between the predicted and measured blood pressure was 0.9 or more.

The significance levels of the tests were set at $p < 0.05$.

**Figure 3:**
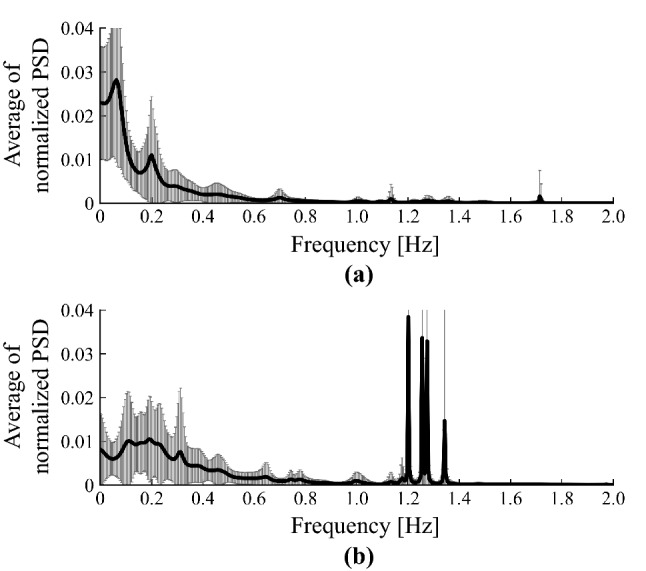
Averages of normalised PSD of peripheral arterial stiffness $\beta$ for all subjects: (**a**) pre SNB, (**b**) post SNB.

#### Frequency spectrum analysis of peripheral arterial stiffness

Power spectral analysis was performed to examined whether the frequency components in peripheral arterial stiffness $\beta$ synchronise with the vital signals as observed in MSNA. In ETS, the surgeon searches for the thoracic sympathetic trunk of the patients, and blocks it using clips. Therefore, stimulation searching for the thoracic sympathetic trunk enhances peripheral sympathetic nerve activity, whereas thoracic sympathetic trunk blockage decreases its activity. Thus, the analysis was set to 30 s before right thoracic sympathetic trunk blocking (hereinafter called “pre SNB”), and 30 s after 60 s had passed after right thoracic sympathetic trunk blocking (hereinafter called “post SNB”). Sections where the coefficient of determination between the estimated blood pressure based on the stiffness $\beta$ and the measured blood pressure was 0.9 or more were chosen for analysis.

Power spectral density (PSD) of peripheral arterial stiffness $\beta$ was calculated using the autoregressive model. The previous study reported that oversampling of the signal does not provide any advantage because it only increases the analysis time and required model order^14^. In order to focus on the frequency band below 2 Hz, the peripheral arterial stiffness $\beta$ estimated at a sampling frequency of 125 Hz was downsampled to 5 Hz after anti-aliasing operation (a low-pass filter with a cutoff frequency of 2 Hz). Thereafter, PSD of the peripheral stiffness $\beta$ was calculated using the Burg method^15^. The order of the autoregressive model was determined based on the minimised Akaike’s information criterion (AIC)^16^. The result of calculating AIC from pre SNB and post SNB was $20.7 \pm 9.8$ (mean ± standard deviation). A previous study regarding heart rate variability with periodic characteristics similar to MSNA reported that the order obtained by AIC is slightly smaller than the optimal order^14^. The order of the autoregressive model was therefore set to 23. The calculated PSD was normalised by dividing with the total power in each analysis interval of each subject.

Cross power spectral density (CPSD) between the peripheral arterial stiffness $\beta$ and systolic blood pressure (SBP) was calculated using a bivariate autoregressive model. The peripheral arterial stiffness $\beta$ was estimated at a sampling frequency of 5 Hz, as well as the PSD calculation. SBP was resampled at 5 Hz using cubic spline interpolation. Then, CPSD was calculated using the Nuttall-Strand method^17^, which is a cross power spectral analysis method. The model order was determined based on Akaike’s information corrected criterion (AICC)^18^. The order was set to 12 because the minimised AICC calculated from pre SNB and post SNB was $11.5 \pm 2.3$. Thereafter, magnitude squared coherence (MSC) between the peripheral stiffness $\beta$ and SBP was calculated. MSC is an index indicating the strength of linear correlation between two signals in a certain frequency band. The significance of MSC between peripheral stiffness $\beta$ and SBP was verified for each subject and each frequency band, using the surrogate data method^19^ with the significance level set at $p < 0.05$.

#### Relationship analysis between $\beta$ and vital signals

The peripheral arterial stiffness $\beta$ was evaluated to verify whether the following MSNA features were present.

(i) Heart rate

The relationship between heart rate and $\beta$ was verified by applying linear regression analysis for the heartbeat frequency and the peak frequency of pre/post SNBs calculated from the electrocardiogram. The heartbeat frequencies were calculated from the average heartbeat intervals, and then the local peak frequency of $\beta$ closest to the heartbeat frequency was extracted. It is assumed that the heartbeat frequency matches the peak frequency of peripheral arterial stiffness $\beta$, the slope of regression line between the heartbeat frequency and the peak frequency of the peripheral arterial stiffness will be 1 and the line will pass through the origin. The significance levels of the tests were set at $p < 0.05$.

**Figure 4:**
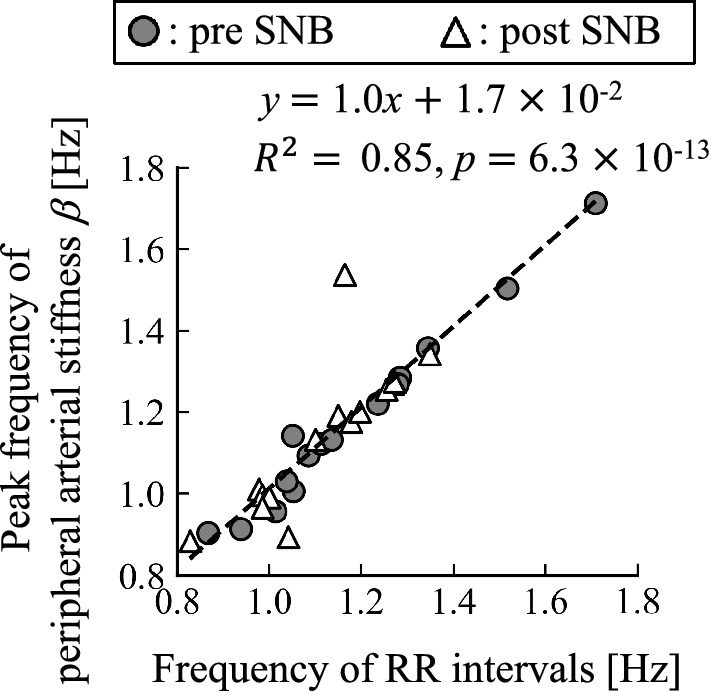
Relationship between peak frequency of peripheral arterial stiffness $\beta$ and that of RR intervals for all subjects.

(ii) Respiratory rate

The relationship between respiratory rate and $\beta$ was investigated by analysing the frequency components corresponding to the respiratory rate. The respiratory rate of the subjects during ETS was controlled at 12 times/min, corresponding to 0.2 Hz. Therefore, if the respiratory component is included in the peripheral arterial stiffness $\beta$, a PSD peak of the peripheral arterial stiffness $\beta$ appears around 0.2 Hz. A previous study reported that the respiratory rate usually measures at 9–24 times/min and synchronises with HF (0.15–0.4 Hz)^20^. The peak frequencies at HF of the peripheral arterial stiffness $\beta$ in pre SNB and post SNB were calculated, in addition the histograms were created by dividing every 0.025 Hz. 95$\%$ confidence interval of the peak frequencies was calculated for investigating that frequency included in 0.2 Hz.

(iii) Systolic blood pressure

The relationship between SBP and $\beta$ was investigated by analysing their MSC. Prior research reported that MSNA and SBP correlated with LF (0.04–0.15 Hz)^21^ because the LF component of MSNA is thought to induce vasomotion by sympathetic nerve activity^22^. Hence, the maximum MSC at LF between the peripheral arterial stiffness $\beta$ and SBP of each subject was examined to verify the significance of pre SNB with enhancing sympathetic nerve activity. Furthermore, the sum for normalised power at LF of the peripheral arterial stiffness $\beta$ in pre SNB was compared with that in post SNB to verify whether LF component of the peripheral arterial stiffness reflects sympathetic nerve activity as well as MSNA. The Wilcoxon signed rank test was used to determine the significance of differences, with the significance level set at $p < 0.05$.

## Results

### Accuracy verification of the estimation of peripheral arterial stiffness

Figure 1 shows the measured biosignals and peripheral arterial stiffness from Sub. A. From top to bottom, each figure shows measured electrocardiograms, continuous arterial pressures, photo plethysmograms, the peripheral arterial stiffness of the proposed $\beta$ and that of the conventional $\beta _{beat}$, and enlarged figure of the peripheral arterial stiffness of the proposed $\beta$ and that of the conventional $\beta _{beat}$. Figure 1 indicates that the blood pressure increased and photoplethysmographic amplitude decreased during right thoracic sympathetic trunk stimulation. The same figure also indicates that the contours of the peripheral arterial stiffness of the proposed $\beta$ and that of the conventional $\beta _{beat}$ match, and the temporal resolution of the peripheral arterial stiffness of the proposed $\beta$ is improved compared to that of the conventional $\beta _{beat}$.

Figure 2 shows that the result of Bland-Altman analysis between the peripheral arterial stiffness of the proposed $\beta$ and that of the conventional $\beta _{beat}$. The 95$\%$ confidence interval obtained from differences between the peripheral arterial stiffness of the proposed $\beta$ and that of the conventional $\beta _{beat}$ was $-5.2\times 10^{-2}$–$5.3\times 10^{-2}$ and constant error was not seen. Proportional error between the peripheral arterial stiffness of the proposed $\beta$ and that of the conventional $\beta _{beat}$ could be seen ($p=5.4\times 10^{-60}$). However, both the correlation coefficient and slope of the regression line between the difference and average of the two indices were small, indicating no bias in the estimation ($r=-0.11$, slope of the regression line $a=-1.2\times 10^{-2}$).

**Figure 5:**
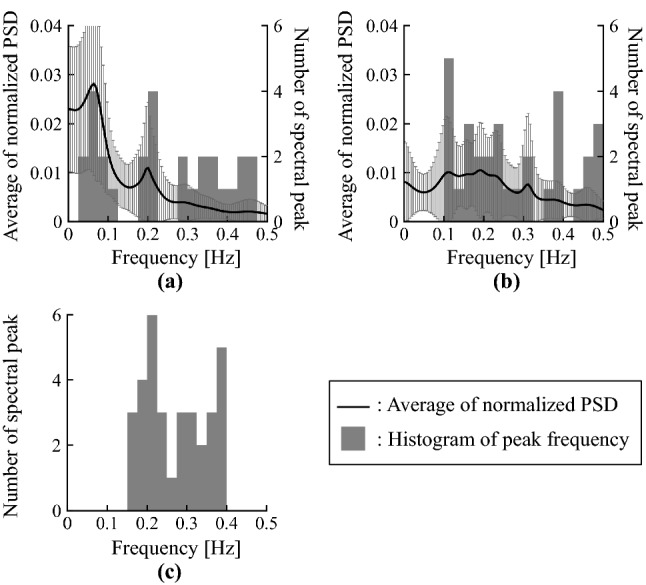
Averages of normalised PSD and histograms of peak frequency for peripheral arterial stiffness $\beta$ for all subjects: (**a**) pre SNB, (**b**) post SNB, (**c**) sum of pre SNB and post SNB measured at high frequency range HF.

### Relationship between vital signals and peripheral arterial stiffness

Figure 3 shows the averages of normalised PSD of peripheral arterial stiffness $\beta$ for all subjects. Figure 3a,b show the results for pre SNB and post SNB, respectively. Figure 3 indicates that there are multiple spectral peaks at the frequency band below 0.5 Hz for both pre SNB and post SNB. In addition, Fig. 3b highlights spectral peaks around 1.2 Hz for post SNB.

Figure 4 shows the relationship between the peak frequencies of peripheral arterial stiffness $\beta$ and that of RR intervals for all subjects. The regression line between them was significant ($R^{2}=0.85$, $p=6.3\times 10^{-13}$) and its slope was 1.0. The 95$\%$ confidence interval for the intercept of the regression line was between − 0.17 and 0.21, and zero was included in that interval.

Figure 5 shows the averages of normalised PSD and histograms of peak frequency for the peripheral arterial stiffness $\beta$ for all subjects. Figure 5a,b show the results for pre SNB and post SNB, respectively. There were spectral peaks around 0.2 Hz corresponding to the respiratory cycle in both pre SNB and post SNB. Figure 5c shows the sum of frequency histogram for the peripheral arterial stiffness $\beta$ of both pre SNB and post SNB measured at HF. The 95$\%$ confidence interval for peak frequencies of the peripheral arterial stiffness $\beta$ included 0.2 Hz.

**Figure 6:**
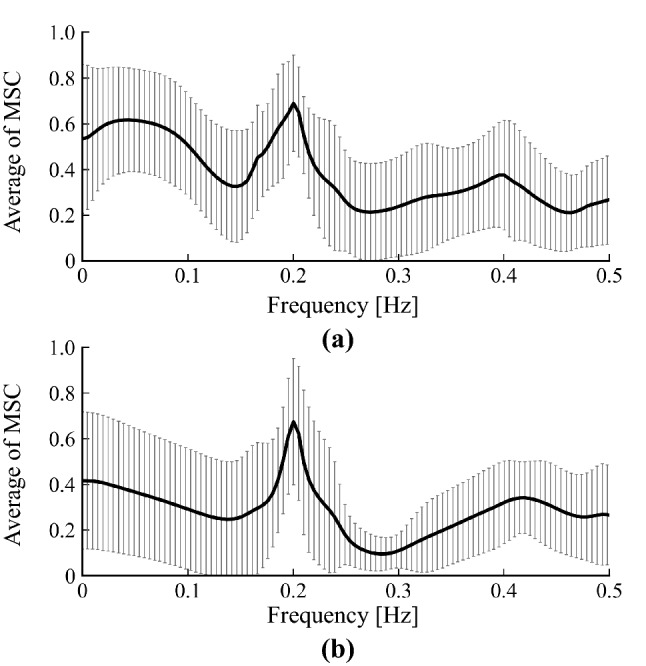
Averages of MSC between peripheral arterial stiffness $\beta$ and SBP for all subjects: (**a**) pre SNB, (**b**) post SNB.

Figure 6 shows the averages of MSC between the peripheral arterial stiffness $\beta$ and SBP for all subjects. Figure 6a,b show the results for pre SNB and post SNB, respectively. There were peaks of the MSC around 0.04 Hz and 0.2 Hz in pre SNB and 0.2 Hz in post SNB.

**Figure 7:**
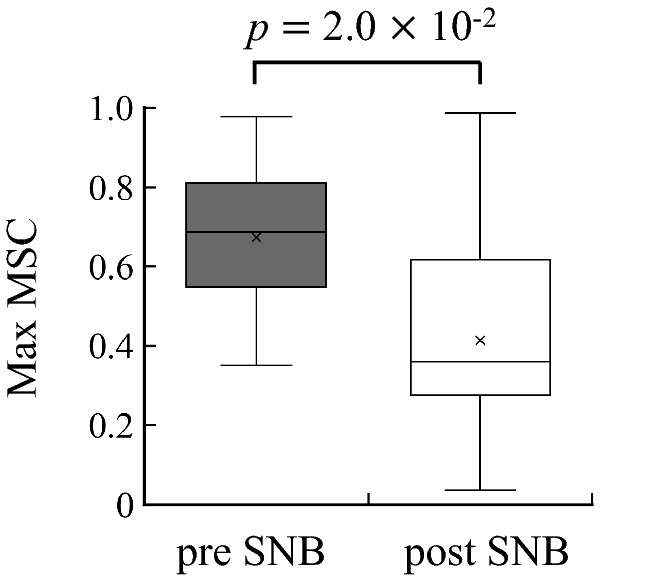
Max MSC at low frequency range LF between peripheral arterial stiffness $\beta$ and SBP before and after sympathetic nerve block by clipping.

Figure 7 shows the maximum MSC at LF between peripheral arterial stiffness $\beta$ and SBP for pre SNB and post SNB. Figure 7 indicates that the average of the maximum MSC is 0.67 and median of that is 0.69 in pre SNB, respectively. Significant values were obtained from 13 of 15 subjects. In addition, the same figure indicates that the average of the maximum MSC is 0.41 and median of that is 0.36 in post SNB, respectively. Significant values were obtained from 8 of 15 subjects. The maximum MSC of pre SNB was significantly greater than that of post SNB ($p=2.0\times 10^{-2}$).

**Figure 8:**
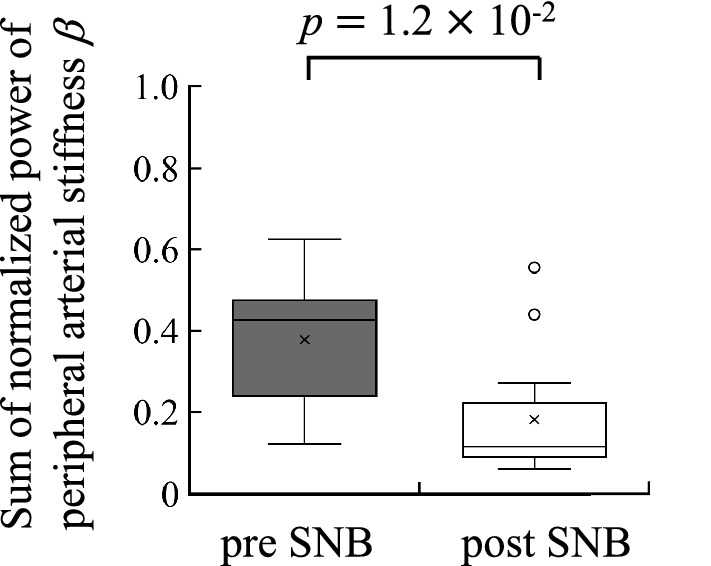
Comparison of sum of normalised power of peripheral arterial stiffness $\beta$ at low frequency range LF before and after sympathetic nerve block by clipping.

Figure 8 shows the comparison results between the sum for normalised power of the peripheral arterial stiffness $\beta$ at LF of pre SNB and that of post SNB. Figure 8 indicates that the sum for normalised power of the peripheral arterial stiffness $\beta$ at LF of pre SNB were significantly greater than that of post SNB ($p=1.2\times 10^{-2}$).

## Discussion

It was confirmed that the peripheral arterial stiffness $\beta$ increased during stimulation of the right thoracic sympathetic trunk as shown in Fig. 1. This indicates sympathetic nerve activation and subsequent contraction of the peripheral arterial walls resulting from stimulation. Meanwhile, the peripheral arterial stiffness $\beta$ did not increase during stimulation of the left thoracic sympathetic trunk. Therefore, the peripheral sympathetic nerve on the right does not respond to stimulation because the right thoracic sympathetic trunk had already been blocked. It was confirmed that the estimation accuracy of the proposed peripheral arterial stiffness $\beta$ was equivalent to that of the conventional peripheral arterial stiffness $\beta _{beat}$ and the temporal resolution of the estimated arterial stiffness was improved. As a result of Bland-Altman analysis, no constant error was found, as shown in Fig. 2. Significant correlation was confirmed between mean values and errors of the proposed peripheral arterial stiffness $\beta$ and the conventional peripheral arterial stiffness $\beta _{beat}$. However, this proportional error is negligible owing to the small slope of the regression line. From the above, the proposed method for estimating peripheral arterial stiffness can improve the temporal resolution and realise equidistant sampling while maintaining high estimation accuracy.

Spectral peaks of the peripheral arterial stiffness $\beta$ were confirmed at the frequency band below 2.0 Hz in both pre SNB and post SNB, as shown in Fig. 3. The features of peripheral arterial stiffness $\beta$ agree with those of MSNA.

First, it was confirmed that the regression line between the heartbeat frequencies and the peak frequencies of the peripheral arterial stiffness $\beta$ was significant, as shown in Fig. 4. The heartbeat frequencies roughly match the peak frequencies of peripheral arterial stiffness $\beta$ because almost all plots are on a straight line with a slope of 1.0 passing through the origin. Therefore, peripheral arterial stiffness $\beta$ synchronises with the heartbeat as MSNA.

Second, it was confirmed that there were spectral peaks of the peripheral arterial stiffness $\beta$ around 0.2 Hz, corresponding to the respiratory cycle, for both pre SNB and post SNB, as shown in Fig. 5a,b. Further, the peaks at HF were concentrated around 0.2 Hz, as shown in Fig. 5c. The peripheral arterial stiffness $\beta$ was considered to synchronise with the respiratory rate because the 95$\%$ confidence interval of the peak frequencies included 0.2 Hz at HF. The spectral peaks around 0.4 Hz were harmonics of the respiratory component. However, the influence of the harmonics was considered to be small, because the averages of normalised PSD for the peripheral arterial stiffness $\beta$ around 0.4 Hz were small in both pre SNB and post SNB. Therefore, peripheral arterial stiffness $\beta$ exhibits feature of MSNA where it synchronises with respiratory cycle.

Finally, the peaks in the average MSC between peripheral arterial stiffness $\beta$ and SBP were found around 0.04 Hz and 0.2 Hz for pre SNB and were found around 0.2 Hz for post SNB (see Fig. 6). The peaks around 0.2 Hz represent the respiratory component, because that component was synchronised with not only the peripheral arterial stiffness $\beta$ but also SBP^23^. Conversely, the MSC at LF showed significant values for most subjects in pre SNB, and it was confirmed that the peripheral arterial stiffness $\beta$ linearly correlated with SBP at LF (see Fig. 7). The MSC for pre SNB displayed significantly large value compared with that for post SNB, and this result was consistent with the previous study^11^. Furlan et al. reported that the MSC at LF between MSNA and SBP during the tilt test was significantly greater than at rest^11^. This result indicated that common periodic oscillations in both MSNA and SBP were induced at LF because of the sympathetic hyperactivity associated with the tilt test^24^. Meanwhile, the power ratio at LF of peripheral arterial stiffness $\beta$ in pre SNB was significantly greater than that in post SNB (see Fig. 8). Hence, the LF component of peripheral arterial stiffness $\beta$ was considered to reflect sympathetic nerve activity as that exhibited by MSNA.

This paper clarified that peripheral arterial stiffness $\beta$ inherits the features of MSNA, with its frequency components pulse-synchronous with heart and respiration rates and correlating with the LF component of SBP. Non-invasive evaluation of MSNA that requires invasive measurement may be realised with the peripheral arterial stiffness $\beta$. However, peripheral arterial stiffness $\beta$ may not only display features of MSNA, but also those of skin sympathetic nerve activity, which plays a role in thermoregulation^25^. The skin sympathetic nervous activity is known to reflect in the photoplethysmogram^26^. Therefore, the effect of skin sympathetic nerve activity on peripheral arterial stiffness $\beta$ has to be investigated to enable non-invasive evaluation of MSNA.

## Supplementary Information

Supplementary Information 1.
