# Supplementary material for: Cardiorespiratory synchronisation and systolic blood pressure correlation of peripheral arterial stiffness during endoscopic thoracic sympathectomy
Source: Sci Rep. 2021 Mar 16;11:5966. doi: 10.1038/s41598-021-85299-y (PMC7966741; doi:10.1038/s41598-021-85299-y)
Supplement: Supplementary file 1 — Supplementary Information 1. [file 41598_2021_85299_MOESM1_ESM.pdf]

Cardiorespiratory Synchronisation and Systolic Blood Pressure Correlation of Peripheral Arterial Stiffness During Endoscopic Thoracic Sympathectomy

Toshifumi Muneyasu[1], Harutoyo Hirano[2], Akira Furui[1], Zu Soh[1], Ryuji Nakamura[3], Noboru Saeki[3], Yoshiyuki Okada[4], Masashi Kawamoto[5], Masao Yoshizumi[6], AtsuoYoshino[7], Takafumi Sasaoka[8], Shigeto Yamawaki[7,8], and Toshio Tsuji[1,*]

1. Graduate School of Advanced Science and Engineering, Hiroshima University, 1-4-1 Kagamiyama,Higashi-Hiroshima, Hiroshima, 739-8527, Japan
2. Academic Institute, College of Engineering, Shizuoka University, 3-5-1, Johoku, Naka-ku, Hamamatsu, Shizuoka,432-8561, Japan
3. Department of Anesthesiology and Critical Care, Graduate School of Biomedical and Health Sciences, HiroshimaUniversity, 1-2-3 Kasumi, Minami-ku, Hiroshima, Hiroshima, 734-8553, Japan
4. Department of Special Care Dentistry, Graduate School of Biomedical and Health Sciences, Hiroshima University,1-2-3 Kasumi, Minami-ku, Hiroshima, Hiroshima, 734-8553, Japan
5. Medical Corporation JR Hiroshima Hospital, 3-1-36 Futabano-sato, Higashi-ku, Hiroshima, Hiroshima, 732-0057, Japan
6. Department of Cardiovascular Physiology and Medicine, Graduate School of Biomedical and Health Sciences,Hiroshima University, 1-2-3 Kasumi, Minami-ku, Hiroshima, Hiroshima, 734-8553, Japan
7. Department of Psychiatry and Neurosciences, Division of Frontier Medical Sciences, Graduate School of Biomedical and Health Sciences, Hiroshima University, 1-2-3 Kasumi, Minami-ku, Hiroshima, Hiroshima, 734-8551, Japan
8. Center of Innovation, KANSEI Innovation Program, Hiroshima University, 1-2-3 Kasumi, Minami-ku,Hiroshima,Hiroshima, 734-8551, Japan

*tsuji-c@bsys.hiroshima-u.ac.jp

Supplemental Information

CALCULTION METHOD FOR A LOG-LINEARIZED PERIPHERAL ARTERIAL VISCOELASTIC INDEX IN A BEAT-TO-BEAT MANNER

In quantitative evaluation of the mechanical characteristics of peripheral arteries, the following three points should be considered: (i) arterial wall mechanical characteristics can be quantitatively evaluated using mechanical impedance parameters, such as stiffness and viscosity; (ii) stiffness changes caused by sympathetic nerve activity can be estimated, considering nonlinearity between intravascular pressure and arterial diameter; and (iii) specific features of peripheral arteries (e.g., effects of veins and accumulation in arterioles) can be expressed. This section introduces a mechanical peripheral arterial wall model that was designed in consideration of these three points.

Figure 1 shows the proposed mechanical impedance model of the peripheral arterial wall[1,2]. The arterial wall's characteristics in an arbitrary radial direction are considered, and are shown with changes in blood pressure and strain of the arterial diameter, as follows:

$$P_b(t) = P_\mu(t) + P_\eta(t) + P_\beta(t)$$
$$\approx \tilde{\mu}\ddot{\epsilon}(t) + \tilde{\eta}\dot{\epsilon}(t) + P_\beta(\epsilon(t)). \quad (1)$$

$P_b(t)$ is the blood pressure at time t; $P_\mu(t)$, $P_\eta(t)$ and $P_\beta(t)$ are the pressures originating from inertia, viscosity, and stiffness, respectively; and $\tilde{\mu}$ and $\tilde{\eta}$ are the arterial wall inertia and viscosity, respectively. The parameters $\epsilon(t)$, $\dot{\epsilon}(t)$, and $\ddot{\epsilon}(t)$ represent the strain of the arterial diameter, strain velocity, and strain acceleration, respectively. Here, we assume that $\tilde{\mu}\ddot{\epsilon}(t)$ and $\tilde{\eta}\dot{\epsilon}(t)$ include an intervascular pressure component that originated from the vein. In addition, considering the nonlinearity between intravascular pressure and the strain, the pressure originating from vein stiffness is expressed as $P_{\tilde{\beta}}\left(\epsilon(t)\right)$. This assumption was verified by the experimental results, as described in our previous publication[3].

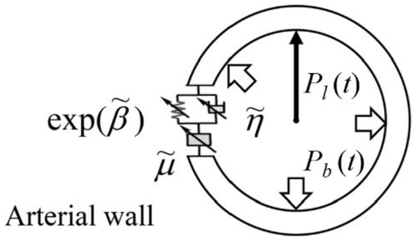

Fig. 1 Mechanical impedance model of a peripheral arterial wall.

$P_{\tilde{\beta}}\left(\epsilon(t)\right)$ is then formulated based on the previous studies and can be viewed as the extended version of the arterial impedance model proposed by Simon *et al.* based on the Voigt model[4,5] through addition of the inertial term and including consideration of the vein effect. The relationship between blood pressure and arterial diameter has been extensively investigated in previous experiments. Nagasawa *et al.* and Hayashi *et al.* measured canine intravascular pressure and the diameter of the arterial wall, using a femoral artery on an *in vitro* basis to support examination of the artery's mechanical properties[6,7]. They found that the canine femoral artery exhibits distensibility in the range of 60–180 mmHg, and that the ratio of the logarithm of intravascular pressure to standard pressure is proportional to the ratio of the arterial wall diameter to its diameter with standard intravascular pressure. However, in the case of low blood pressure, the artery stiffens because the variation in blood vessel diameter, which accompanies increased intravascular pressure, becomes small[6]. Because of this fact, we assumed that human peripheral arterial characteristics are also nonlinear[1,2], as observed in the canine femoral artery, in terms of arterial wall mechanical characteristics. The logarithm pressure originating from arterial stiffness, considering accumulation in arterioles in the peripheral area, is expressed as follows:

$$\ln P_{\tilde{\beta}}\left(\epsilon(t)\right) = \tilde{\beta}\left(\epsilon(t)\right) + P_{\tilde{\beta}_0} + P_{\tilde{\beta}_{nl}}\left(\epsilon(t)\right). \quad (2)$$

$\tilde{\beta}$ is the arterial stiffness relating to logarithm pressure, and $P_{\tilde{\beta}_0}$ is the constant pressure acting on the arterial wall. $P_{\tilde{\beta}_{nl}}\left(r(t)\right)$ is a pressure originating from vein stiffness that cannot be log-linearized and accrues when intravascular pressure falls below a certain threshold. Equation (2) can be substituted into Equation (1) after taking the exponent on both sides of Equation (2), and arterial impedance properties can be expressed as follows:

$$P_b(t) \approx \tilde{\mu}\,\ddot{\epsilon}(t) + \tilde{\eta}\,\dot{\epsilon}(t) + \exp\left\{\tilde{\beta}\epsilon(t) + P_{\tilde{\beta}_0} + P_{\tilde{\beta}_{nl}}\left(\epsilon(t)\right)\right\}. \quad (3)$$

Because it is difficult to measure the strain $\epsilon(t)$ directly *in vivo*, the strain is approximated using PPGs, which are measured with a pulse oximeter[8,9].

$$P_l(t) \cong k_p\epsilon(t) + P_{l_0}. \quad (4)$$

$P_l(t)$ is the PPG, k_p is a constant of proportion, and P_{l_0} is an offset constant. Equation (3) can be expressed using Equation (4), as follows:

$$P_b(t) = \mu\ddot{P}_l(t) + \eta\dot{P}_l(t) + \exp\left\{\beta P_l(t) + P_{b\beta_0} + P_{b\beta_{nl}}\big(P_l(t)\big)\right\}, \quad (5)$$

where

$$\mu = \frac{\tilde{\mu}}{k_p}, \eta = \frac{\tilde{\eta}}{k_p}, \beta = \frac{\tilde{\beta}}{k_p}, P_{b\beta_0} = F_{\tilde{\beta}_0} - \frac{\tilde{\beta}P_{l_0}}{k_p},$$

and $P_{b\beta_{nl}}\big(P_l(t)\big) = P_{\tilde{\beta}_{nl}}\big(\epsilon(t)\big)$ represents blood pressure, which cannot be log-linearized and accrues when intravascular pressure decreases. The parameters μ, η, and β are the inertia, viscosity, and stiffness of the arterial wall, respectively. Equation (5) represents the log-linearized peripheral arterial viscoelastic model, which expresses peripheral arterial dynamic characteristics.

The method for estimating the impedance parameters inertia μ, viscosity η, and stiffness β, is outlined as follows. Estimating three impedance parameters at the same time is difficult because of the characteristic of $P_{b\beta_{nl}}\big(P_l(t)\big)$, which cannot express linearity, even if $P_{b\beta_{nl}}\big(P_l(t)\big)$ is used for the logarithm. Accordingly, estimation of parameters is performed in two stages.

In the first stage, the stiffness component of Equation (5) is approximated by the first-order term function by Maclaurin series expansion, based on the presumption that second- and higher-order terms are sufficiently small:

$$\exp\left\{\beta P_l(t) + P_{b\beta_0} + P_{b\beta_{nl}}\big(P_l(t)\big)\right\} \approx \exp\left\{P_{b\beta_0} + P_{b\beta_{nl}}(0)\right\} + \beta_A P_l(t), \quad (6)$$

The following are details of Maclaurin series expansion: let

$$f(P(t)) = \exp\left\{\beta P_l(t) + P_{b\beta_0} + P_{b\beta_{nl}}\big(P_l(t)\big)\right\}. \quad (7)$$

if the second- and higher-order terms are negligible, then

$$f\big(P(t)\big) \approx f(0) + \frac{df(0)}{dP_l(t)}P_l(t).$$

Since

$$f(0) = \exp\left\{P_{b\beta_0} + P_{b\beta_{nl}}(0)\right\},$$

$$\beta_A = \frac{df(0)}{dP_l(t)} = C_1 \exp\left\{P_{b\beta_0} + P_{b\beta_{nl}}(0)\right\},$$

$$C_1 = \beta + \left.\frac{dP_{b\beta_{nl}}\big(P_l(t)\big)}{dP_l(t)}\right|_{P_l(t)=0}.$$

Therefore, Equation (5) can be approximated as follows:

$$P_b(t) \approx \mu\ddot{P}_l(t) + \eta\dot{P}_l(t) + \exp\left\{P_{b\beta_0} + P_{b\beta_{nl}}(0)\right\} + \beta_A P_l(t). \quad (8)$$

By using variations of the variables from the nominal values at time t_0, the dynamic characteristics of the artery can be expressed as follows:

$$dP_b(t) = \mu d\ddot{P}_l(t) + \eta d\dot{P}_l(t) + \beta_A dP_l(t), \tag{9}$$

where $dP_b(t) = P_b(t) - P_b(t_0)$, $d\ddot{P}_l(t) = \ddot{P}_l(t) - \ddot{P}_l(t_0)$, $d\dot{P}_l(t) = \dot{P}_l(t) - \dot{P}_l(t_0)$, and $dP_l(t) = P_l(t) - P_l(t_0)$. The parameters μ, η, and β_A are then estimated, using the least-squares method for each heartbeat, via Equation (9). β_A is an approximation of the stiffness characteristics.

In the second stage, substituting the estimated inertia $\hat{\mu}$ and viscosity $\hat{\eta}$ in the first stage into Equation (6) yields an equation that can be used to separate the stiffness component from other components, as follows:

$$\exp\left\{\beta P_l(t) + P_{b_{\beta_0}} + P_{b_{\beta_{nl}}}\big(P_l(t)\big)\right\} = P_b(t) - \hat{\mu}\ddot{P}_l(t) - \hat{\eta}\dot{P}_l(t). \tag{10}$$

Taking the exponent on both sides of Equation (10), the following equation can be obtained:

$$\beta P_l(t) + P_{b_{\beta_0}} + P_{b_{\beta_{nl}}}\big(P_l(t)\big) = \ln\{P_b(t) - \hat{\mu}\ddot{P}_l(t) - \hat{\eta}\dot{P}_l(t)\}. \tag{11}$$

By using variations of the variables from the nominal values at time t_0 in the same manner as in Equation (9), Equation (11) can be expressed as follows:

$$\beta dP_l(t) + P_{b_{\beta_{nl}}}\big(P_l(t)\big) - P_{b_{\beta_{nl}}}\big(P_l(t_0)\big) = \ln\left\{\frac{P_b(t)-\hat{\mu}\ddot{P}_l(t)-\hat{\eta}\dot{P}_l(t)}{P_b(t_0)-\hat{\mu}\ddot{P}_l(t_0)-\hat{\eta}\dot{P}_l(t_0)}\right\} \tag{12}$$

Estimation of the stiffness parameter β is limited to the area where $P_{b_{\beta_{nl}}}\big(P_l(t)\big) = P_{b_{\beta_{nl}}}\big(P_l(t_0)\big)$. When the arterial pressure $P_b(t)$ falls below a threshold pressure TH and the artery is considerably stiff, the above condition is not fulfilled. Therefore, the stiffness parameter β is estimated under the area $P_b(t)$ > TH. Equation (12) is then approximated as follows, because $P_{b_{\beta_{nl}}}\big(P_l(t)\big) - P_{b_{\beta_{nl}}}\big(P_l(t_0)\big)$ is equal to 0.

$$\beta dP_l(t)|_{P_b(t)>\mathrm{TH}} = \ln\left\{\frac{P_b(t)-\hat{\mu}\ddot{P}_l(t)-\hat{\eta}\dot{P}_l(t)}{P_b(t_0)-\hat{\mu}\ddot{P}_l(t_0)-\hat{\eta}\dot{P}_l(t_0)}\right\}\Bigg|_{P_b(t)>\mathrm{TH}}. \tag{13}$$

Because the stiffness parameter β in the left side of the above equation is the only unknown parameter in Equation (13), it can be estimated for each heartbeat, using the least-squares method, via Equation (13).

As mentioned above, the impedance parameters (i.e., inertia μ, viscosity η, and stiffness β) of the peripheral arterial wall can be estimated using the two-stage method.

REFERENCES

1. Hirano, H., *et al.* A Log-linearized peripheral arterial viscoelastic index and its application to endoscopic thoracic sympathectomy. *Trans. Soc. Instr. Control Engineers* **48**, 731–739 (2012).

2. Hirano, H., *et al.* Monitoring of peripheral vascular condition using a log-linearized arterial viscoelastic index during endoscopic thoracic sympathectomy. *Conf. Proc. IEEE. Eng. Med. Biol. Soc.* **2013**, 2587–2590 (2013).

3. Matsubara, H. *et al.* Quantitative evaluation of pain during electrocutaneous stimulation using a log-linearized peripheralarterial viscoelastic model. Sci. Rep. 8, 1-9 (2018).

4. Armentano, R.L., Barra, J.G., Levenson, J., Simon, A. & Pichel, R.H. Arterial wall mechanics in conscious dogs: assessment of viscous, inertial, and elastic moduli to characterize aortic wall behavior. *Circ. Res.* **76**, 468–478 (1995).

5. Barra, J.G., *et al*. In vivo angiotensin angiotensin II receptor blockade and converting enzyme inhibition on canine aortic viscoelasticity. *Am. J. Physiol.* **272**, H859–H868 (1997).
6. Nagasawa, S., *et al*. Mechanical properties of canine femoral artery smooth muscle. *J. Jpn. Coll. Angiology*. **20**, 221–226 (1980).
7. Hayashi, K., Handa, H., Nagasawa, S., Okumura, A. & Moritake, K. Stiffness and elastic behavior of human intracranial and extracranial arteries. *J. Biomechanics* **13**, 175–184 (1980).
8. Aoyagi, T. & Ukawa, T. Pulse oximeter which the optical measurement produced. Structure and principle of a pulse oximeter. *Clin. Eng*. **7**, 102–110 (1996).
9. Ukawa, T. Pulse oximeter. *J. Med. Lab*. **22**, 11–16 (1994).
